# Differences in the Gut Microbiota Composition and Metabolites Associated With Feeding Intolerance in VLBW Infants With a Gestational Age of ≤ 30 Weeks: A Pilot Study

**DOI:** 10.3389/fcimb.2022.726322

**Published:** 2022-02-17

**Authors:** Xiao-Chen Liu, Qian Sun, Yan-Chun Ji, Li-Zhen Fu, Zheng-Li Wang, Yu He, Lu-Quan Li

**Affiliations:** ^1^ Neonatal Diagnosis and Treatment Centre of Children’s Hospital of Chongqing Medical University, Chongqing, China; ^2^ National Clinical Research Center for Child Health and Disorders, Chongqing, China; ^3^ Ministry of Education Key Laboratory of Child Development and Disorders, Chongqing, China; ^4^ China International Science and Technology Cooperation base of Child Development and Critical Disorders, Chongqing, China; ^5^ Chongqing Key Laboratory of Pediatrics, Chongqing, China

**Keywords:** feeding intolerance, gut microbiota, short-chain fatty acids (SCFAs), autoinducer-2 (AI-2), metabolites

## Abstract

**Objective:**

To explore the main variations in gut microbiota compositions, short-chain fatty acids (SCFAs) concentrations and autoinducer-2 (AI-2) levels in very-low-birth-weight (VLBW) infants with feeding intolerance (FI).

**Methods:**

Twenty-seven VLBW infants with gestational ages of ≤30 weeks were divided into the FI group (n=14) and feeding tolerance (FT) group (n=13). The gut microbiota composition and SCFAs concentrations and AI-2 levels in feces were detected at 2 and 4 weeks after birth.

**Results:**

There was no difference in alpha diversity between the two groups at 2 and 4 weeks after birth (*P*>0.05). Although the *Chao* index decreased (*P*<0.05), there was no difference in the *Shannon* index from 2 weeks to 4 weeks in either the FI or FT group (*P*>0.05). Additionally, there was no difference in beta diversity between the FI and FT groups at 2 weeks (*P*>0.05), but there was a significant difference in beta diversity between the two groups at 4 weeks (*P*<0.05) and a large difference from 2 weeks to 4 weeks in both the FI and FT groups (*P*<0.05). Furthermore, the composition of the microbiota at 4 weeks was significantly different from that at 2 weeks in the FI group (*P*<0.05). The *Veillonella* abundance was lower at 4 weeks in the FI group (*P*<0.05), but there were no differences in the compositions of the other main microbes between the two groups (*P*>0.05). *Proteobacteria* and *Firmicutes* were dominant in both the FI and FT groups. The concentrations of propanoic, valeric and hexanoic acids were lower in the FI group at 2 weeks, and the levels of isobutyric and valeric acids were lower at 4 weeks after birth (*P*<0.05). The areas under the curves (*AUCs*) of propanoic, butanoic and valeric acids in predicting FI were 0.878, 0.816 and 0.744, respectively. Compared with that in the FT group, the relative bioluminescence of AI-2 was lower in the FI group at 2 weeks (*P*<0.05), and the *AUC* was 0.736.

**Conclusions:**

The main composition of the microbiota was not obviously different in infants with FI. Some SCFAs and AI-2 have moderate value in predicting FI.

## Introduction

Feeding intolerance (FI) in neonates is characterized by abdominal distention, vomiting and a large gastric residual volume after enteral feeding, and the incidence of FI among neonates, especially very or extremely preterm infants, is high (Moore and Wilson, 2011; Eveleens et al., 2020; Reintam Blaser et al., 2021). With prolonged parenteral nutritional support, the risks of nosocomial infections and delays in hospital discharge caused by FI increase (Moore and Wilson, 2011; Chi et al., 2019; Wang et al., 2019; Eveleens et al., 2020; Heyland et al., 2021). These infections may even progress to severe diseases, such as late-onset sepsis (LOS) and necrotizing enterocolitis (NEC) (Chi et al., 2019; Wang et al., 2019; Meister et al., 2020; Ali et al., 2021). Although the incidence of FI is high, and its outcome is poor, the pathophysiology of FI is still unclear.

In preterm neonates, the reduced diversity of the gut microbiota and the prevalence of pathogenic bacteria (e.g., *Klebsiella pneumoniae* and *Clostridium difficile*) might be important causes or main features of many diseases (Chong et al., 2018). Studies have reported that dysbacteriosis in the gut is an important feature of some serious gastrointestinal diseases, such as NEC (Warner et al., 2016; Pammi et al., 2017). Yuan et al. characterized the changes in the microbiota composition in infants with FI and speculated that *Klebsiella* might be a potential biomarker (Yuan et al., 2019). Short-chain fatty acids (SCFAs), organic monocarboxylic acids with a chain length of up to six carbon atoms (Cummings et al., 1987), are the major products of intestinal microbes, and acetate, propionate, and butyrate make up the majority of SCFAs. As the main metabolites of the gut microbiota, SCFAs vary with gut microbiota composition (Liu et al., 2020). Autoinducing molecule-2 (AI-2), also called the “public language” between microbes, is related to the virulence and pathogenicity of bacteria and plays an important role in gut colonization by altering the communication between microbes (Waheed et al., 2020; Zhang et al., 2020). Our preliminary study on AI-2 and NEC found that AI-2 levels changed before alterations in the microbiota were detected, and it was reported that increased AI-2 levels altered the composition of the microbiota affected by antibiotics (Thompson et al., 2015; Fu et al., 2020). Thus, variations in AI-2 levels may affect the gut microbiota. However, no study has explored changes in the microbiota composition, SCFAs concentrations and AI-2 levels in VLBW infants with FI.

This case–control study of 27 VLBW infants with or without FI was performed to evaluate changes in the gut microbiota composition at different levels, and SCFAs concentrations and AI-2 levels were also assessed. We aimed to explore differences in the gut microbiota composition and SCFAs concentrations in infants with FI and identify a potential biomarker that can be used to identify FI earlier.

## Subjects and Methods

### Inclusion and Exclusion Criteria

This study was approved by the Ethics Committee of the Children’s Hospital Affiliated with Chongqing Medical University (No. 2021-67). Parents of the neonates enrolled in this trial signed informed consent forms. This prospective case–control study enrolled VLBW infants admitted to the Neonatal Diagnosis and Treatment Center of Children’s Hospital of Chongqing Medical University. Neonates with gestational ages of less than 30 weeks, birth weights below 1500 g, and births within 24 hours were enrolled in our study. Those who met the following criteria were excluded: (1) death during hospitalization; (2) development of NEC (Bell stage II or above) (Walsh and Kliegman, 1986) or the presence of food-protein-induced enteritis or lactose intolerance that was thought to alter the gut microbiota definitively during the research period; (3) the presence of congenital gastrointestinal malformations (such as congenital intestinal atresia, Hirschsprung disease, poor intestinal rotation, etc.); (4) an incomplete gut microbiota, AI-2 or SCFAs assessment; or (5) lack of consent to participate in this study.

### Diagnostic Criteria

The enrolled infants were divided into two groups: those who met the criteria for FI were included in the FI group, and the others were included in the feeding tolerance (FT) group. The criteria for FI (Moore and Wilson, 2011) were as follows: (1) a gastric residual volume of more than 50% of the previous feeding volume; (2) emesis or abdominal distention or both; and (3) decreased, delayed, or discontinued enteral feeding. Infants enrolled in the FI group met all three criteria.

### Clinical Treatment

Infants enrolled in this study were diagnosed and treated routinely in accordance with the Canadian guidelines for feeding VLBW infants (Dutta et al., 2015). Those with a high risk of early-onset sepsis were treated with penicillin after birth, and the course was based on blood culture (Polin, 2012). Other treatments, including parental nutrition and intensive care (respiratory support, blood or blood product transfusion), were performed when necessary.

### Data Collection

Relevant clinical data, including baseline information, diseases and medication during pregnancy, delivery model, feeding and treatment during hospitalization, and hospital outcome, were collected.

### Fecal Sampling

Fresh fecal samples were collected with disposable sterile swabs from the diapers of all the infants daily. The samples were transferred and aliquoted into 1.5-ml sterile, enzymatic EP tubes, with 250 mg per tube, and stored in a freezer at -80°C. We found that all cases of FI occurred within 2 weeks after birth and that all symptoms had resolved at 4 weeks. Therefore, fecal samples collected at 2 and 4 weeks of life in the FI and FT groups were examined.

### DNA Extraction, PCR Amplification and High-Throughput Sequencing

Fecal microbial genomic DNA was extracted from the fecal sample with a QIAamp FAST DNA Stool Mini-Kit (Qiagen, Germany). DNA extracts were detected in 1% agarose gels, and the concentration and purity of the DNA were evaluated with a spectrophotometer (NanoDrop 2000 UV–vis, Thermo Scientific, USA). The V3–V4 hypervariable regions of the 16S rDNA gene were amplified with the primers 338F (5’-ACTCCTACGGGAGGCAGCAG-3’) and 806R (5’-GGACTACHVGGGTWTCTAAT-3’). The cycle was as follows: (1) initial denaturation for 3 minutes at 95°C; (2) denaturation for 30 seconds at 95°C; (3) annealing for 30 seconds at 55°C; and (4) extension for 45 seconds at 72°C. The steps were performed for 27 cycles, with a final extension for 10 minutes at 72°C. The products were extracted with 2% agarose gel electrophoresis, recovered with an AxyPrep DNA Gel Extraction Kit (Axygen Biosciences, USA) and quantified with a Quantus Fluorometer (Promega, USA). Finally, amplicons were pooled in equimolar amounts and paired-end sequenced (2×250) on the Illumina MiSeq platform (California, USA) according to standard protocols. The reads were distinguished based on primers and barcodes, and the sequence direction was adjusted to ensure accurate barcode matching.

### Bioinformatic Analysis

The original data of the microbiota was processed with QIIME (version 1.9.1; Colorado, USA). In brief, bases with a quality score of less than 20 were truncated, and sequences with a length greater than 10 bp were overlapped. Reads exceeding the maximum mismatch ratio of 0.2 in the overlapping regions of the spliced sequences were removed. The sequences were divided into operational taxonomic units (OTUs) with UPARSE (version 7.0.1090; California, USA), and the OTUs were clustered with a similarity threshold of 97% (Amato et al., 2013).

### SCFAs Measurements

The fecal samples were ground twice for 3 minutes, placed in an ice bath for 30 minutes, held at 4°C for 30 minutes and centrifuged at 4°C for 15 minutes. Then, the supernatant was added to ethyl acetate for extraction, incubated in an ice bath for 10 minutes, and centrifuged at 4°C for 10 minutes. Additionally, an appropriate amount of ethyl acetate was sequentially added to 8 SCFAs (including acetic, propionic, butyric, isobutyric, valeric, isovaleric, hexanoic and isohexanoic acids) and 2-ethylbutyric acid to obtain a mixed standard stock solution containing the 8 SCFAs and an internal standard stock solution. The latter was diluted with the SCFAs solution to different concentrations. The concentrations of acetic, propionic and butyric acids ranged from 0.2 to 400 μg/ml, while those of isobutyric, valeric, isovaleric, hexanoic and isohexanoic acids ranged from 0.1 to 200 μg/ml. The processed sample and the standard solution sample were subjected to gas chromatography–mass spectrometry (GC–MS) detection and analysis (Sangster et al., 2006; Ponnusamy et al., 2011; Ng et al., 2012). Finally, we used Masshunter quantitative software (version10.0.707.0; Palo Alto, USA) to automatically identify and integrate target SCFAs. The SCFAs concentrations of each sample were calculated by standard curves.

### AI-2 Measurement

Each 40-mg fecal sample was mixed with 1.6 ml of 2216E (QDRS BIOTEC, China) broth, vortexed for 5 minutes, and centrifuged at 5,000 rpm for 10 minutes at 4°C to obtain test samples. The *V. harveyi* BB170 strain (obtained from Prof. Baolin Sun, University of Science and Technology, China) was cultivated for 18 hours at 30°C in 2216E medium, diluted at a 1:5,000 ratio in fresh 2216E medium and centrifuged at 14,000 rpm for 10 minutes at 4°C. Then, the samples were filtered through a 0.22-µl filter membrane and stored at -20°C. The filtrate was harvested and added to the *V. harveyi* BB170 strain diluent for the AI-2 bioassay (Omm Scientific, USA). Then, 180 µl of the BB170 dilution with 20 µl of fecal filtrate was added in quintuplicate to a 96-well assay plate (Corning, USA), and the plate was agitated at 120 rpm at 30°C. After 2.5 hours, bioluminescence intensity was measured every 30 minutes by a multifunction microplate reader (Thermo, USA) until the value of the control group was minimized. The ratio of the fluorescence of the fecal sample supernatant to that of the 1-µmol AI-2 standard solution represented the relative luminescence intensity.

### Data Analysis

All the clinical, AI-2 and SCFAs data were analyzed with SPSS statistical software (version 23; Chicago, USA). Normally distributed measurement data are presented as means ± S. D and were analyzed by *Student’s t test*. Nonnormally distributed measurement data are presented as medians (interquartile ranges, IQRs) and were analyzed by the *Wilcoxon rank-sum test*. Count data were analyzed by *Fisher’s exact test*. Comparisons of gut microbiota compositions between different groups were analyzed by the *Wilcoxon rank-sum test*. Beta diversity was compared by using principal coordinates analysis (*PCoA*) based on the selected distance matrix, and correlation heatmap analysis was conducted to show the relationship between SCFAs concentrations and the microbiota composition based on *Spearman rank correlation* in R (version 3.3.1; Auckland, New Zealand). In addition, we used the linear discriminant analysis (LDA) effect size (*LEfSe*) obtained with the *Kruskal*–*Wallis (KW) rank-sum test* to further describe variations among microbial groups. Receiver operating characteristic (ROC) curves and all figures were generated with GraphPad Prism (version 9.0; California, USA).

## Results

During the research period, 29 VLBW infants were enrolled in the study; 2 were excluded from further study due to NEC, and 27 infants were finally included in the study. Among them, 13 were included in the FT group, and 14 were included in the FI group. Forty-nine fecal samples were collected and measured at 2 and 4 weeks.

### Clinical Features

All the enrolled infants received breast milk and were fed donor milk when enough breast milk could not be obtained. Compared with the FT group, the FI group had longer parenteral nutritional support and hospitalization times (*P*<0.05). There were no significant differences in the other factors, such as delivery methods, diseases during pregnancy, antibiotic courses or outcomes (*P*>0.05) (**Table 1**).

**Table 1:** Clinical features of the infants enrolled in this study.

	FT (n=13)	FI (n=14)	*Χ^2^*/*Z*/*t*	*P*
Male, % (n)	46.2 (6)	57.1 (8)	/	0.427
Admission age, median (IQR), h	2.00 (1.00-29.43)	1.78 (1.00-2.25)	0.399	0.528
Gestational age, median (IQR), w	29.14 (28.07-29.43)	28.93 (27.61-29.50)	0.216	0.642
Birth weight, ¯x± S. D, g	1173.85 ± 177.132	1110.14 ± 199.943	0.874	0.391
Cesarean section, % (n)	53.8 (7)	64.3 (7)	/	0.436
PROM, % (n)	38.5 (5)	28.6 (4)	/	0.445
Intrauterine distress, % (n)	7.7 (1)	21.4 (3)	/	0.327
Maternal hypertension, % (n)	7.7 (1)	28.6 (4)	/	0.186
Gestational diabetes mellitus, % (n)	38.5 (5)	21.4 (3)	/	0.293
Antenatal steroid exposure, % (n)	76.9 (10)	64.3 (9)	/	0.385
Apgar 1 minute, ¯x± S. D	7.15 ± 1.519	6.14 ± 2.568	1.232	0.229
Apgar 5 minutes, ¯x± S. D	8.62 ± 1.261	8.21 ± 1.188	0.851	0.403
Age of FI, ¯x± S. D, d	/	13.07 ± 5.136	/	/
Weight gain, ¯x± S. D, g				
14 d	195.91 ± 74.021	149.36 ± 117.420	1.112	0.279
28 d	615.50 ± 188.819	484.82 ± 138.233	1.822	0.084
Antibiotic course, median (IQR), d	7.00 (4.00,10.50)	11.00 (6.00,18.00)	1.824	0.068
PICC, % (n)	100.0 (13)	92.9 (13)	/	0.519
PICC period, median (IQR), d	24.00 (19.50-25.50)	22.00 (17.50-23.00)	2.641	0.104
Endotracheal intubation, % (n)	42.6 (6)	57.1 (8)	/	0.427
Endotracheal intubation course, median (IQR), d	4.00 (2.75-5.00)	5.5 (3.25-15.25)	1.305	0.192
Milking age, median (IQR), h	0.00 (0.00-1.00)	1.00 (0.00-2.50)	2.089	0.148
Infants receiving donor milk, % (n)	100.0 (13)	78.6 (11)	/	0.222
Feeding amount, ml/3 h				
14 d, ¯x± S. D, ml/3 h	19.92 ± 3.753	13.85 ± 5.475	3.206	0.004
28 d, median (IQR), ml/3 h	36.00 (33.25-37.75)	25.00 (10.00-36.50)	6.314	0.012
Fasting, % (n)	30.8 (4)	78.6 (11)	/	0.017
Pumping feeding, % (n)	30.8 (4)	71.4 (10)	/	0.035
Parenteral nutrition courses, median (IQR), d	25.00 (22.00,28.00)	28.00 (28.00-28.00)	1.704	0.004
hospital stays, ¯x± S. D, d	56.15 ± 12.562	71.29 ± 16.297	2.686	0.013

FI, feeding intolerance; FT, feeding tolerance; S. D, Standard Deviation; IQR, interquartile range; PROM, premature rupture of membranes >18 h; PICC, peripherally inserted central catheter.

### Microbiota Characteristics

**Figure 1:**
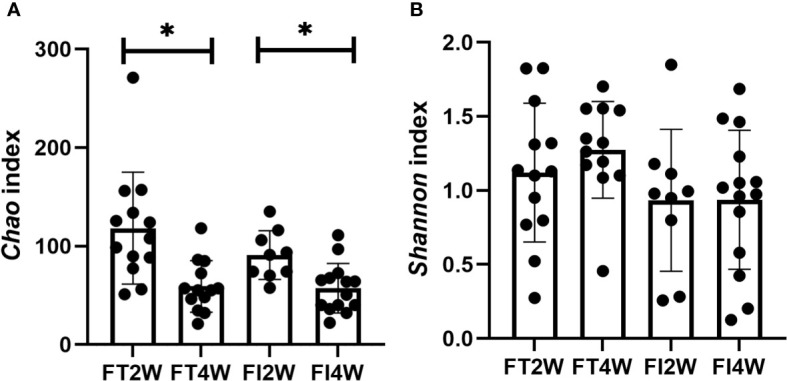
Alpha diversity of the microbiota in the different groups. There were no differences in the *Chao* index between the two groups at 2 and 4 weeks after birth, and it decreased from 2 to 4 weeks in both the FT and FI groups **(A)**. There was no difference in the *Shannon* index between the FI and FT groups or within the FI or FT group at 2 and 4 weeks of life **(B)**. *:*P*<0.05.

In our comparison of alpha diversity, there was no difference in the *Chao* or *Shannon* index between the two groups at 2 or 4 weeks after birth (*P*>0.05). Within-group comparisons at 2 and 4 weeks showed that the *Chao* index decreased (*P*<0.05), but there was no difference in the *Shannon* index in either the FT or FI group (*P*>0.05) **(** **Figure 1** **)**.

**Figure 2:**
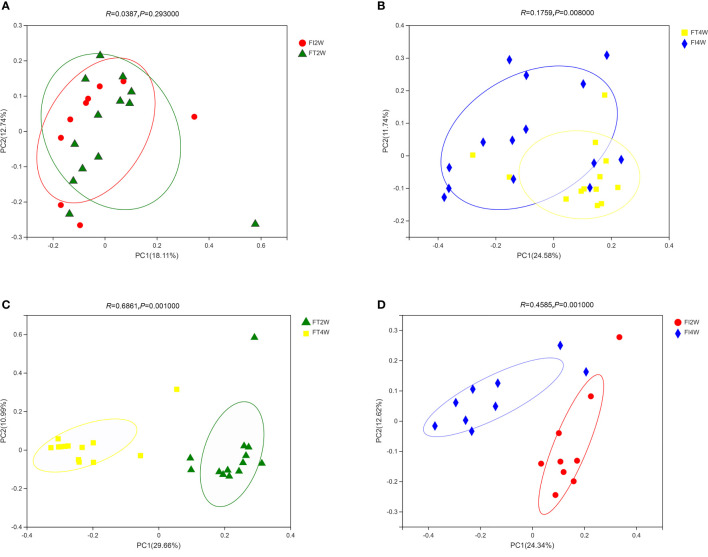
Beta diversity of the microbiota in the different groups. There was no difference in beta diversity between the two groups at 2 weeks **(A)**, but there was a significant difference between the two groups at 4 weeks (*P*<0.05) **(B)**. The beta diversity of the microbiota at 4 weeks was significantly different from that at 2 weeks in the FT **(C)** and FI groups **(D)**.

In our comparison of beta diversity, there was no difference between the FI and FT groups at 2 weeks (*P*>0.05); however, there was a significant difference between the two groups at 4 weeks (*P*<0.05). Additionally, the composition of the microbiota at 4 weeks was significantly different from that at 2 weeks in the FI group (*P<0.05*). A similar result was found in the FT group **(** **Figure 2** **)**.

**Figure 3:**
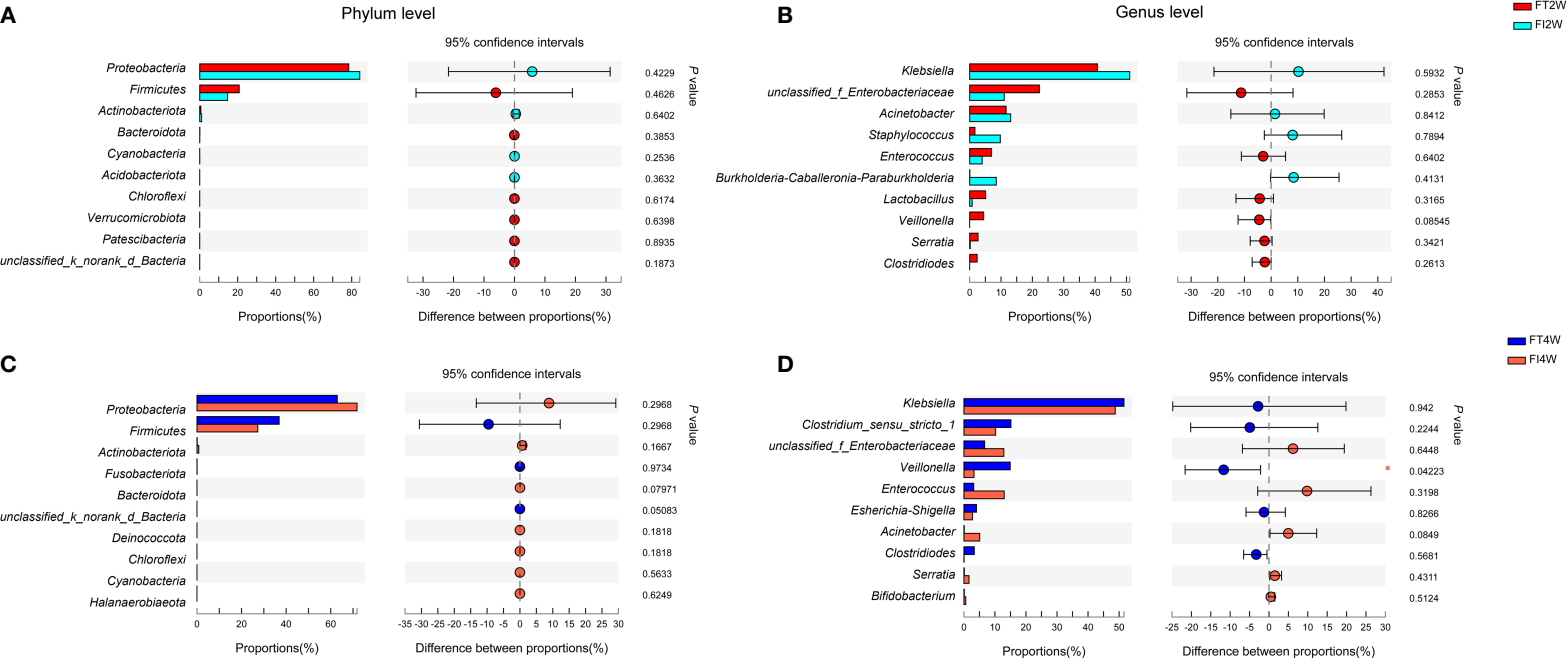
Community abundance in the FI and FT groups. There were no differences between the two groups at the phylum level **(A)** or genus level **(B)** at 2 weeks of life. There were no differences between the two groups at the phylum level at 4 weeks after birth **(C)**. The abundance of *Veillonella* at the genus level was much lower in the FI group than in the FT group at 4 weeks of life **(D)**.

We further compared the main microbiota between the FI and FT groups and found that there was no significant difference between the two groups at the phylum level at 2 or 4 weeks after birth (*P*>0.05). *Proteobacteria* and *Firmicutes* were dominant in both the FI and FT groups. At the genus level, the composition of the microbiota was not different between the two groups at 2 weeks (*P*>0.05), and it was dominated by *Klebsiella*. *Veillonella* was less abundant in the FI group (*P*<0.05) at 4 weeks than at 2 weeks **(** **Figure 3** **)**, and it scored above 4 in the *LEfSe* analysis **(** **Supplementary Figure S1** **)**.

**Figure 4:**
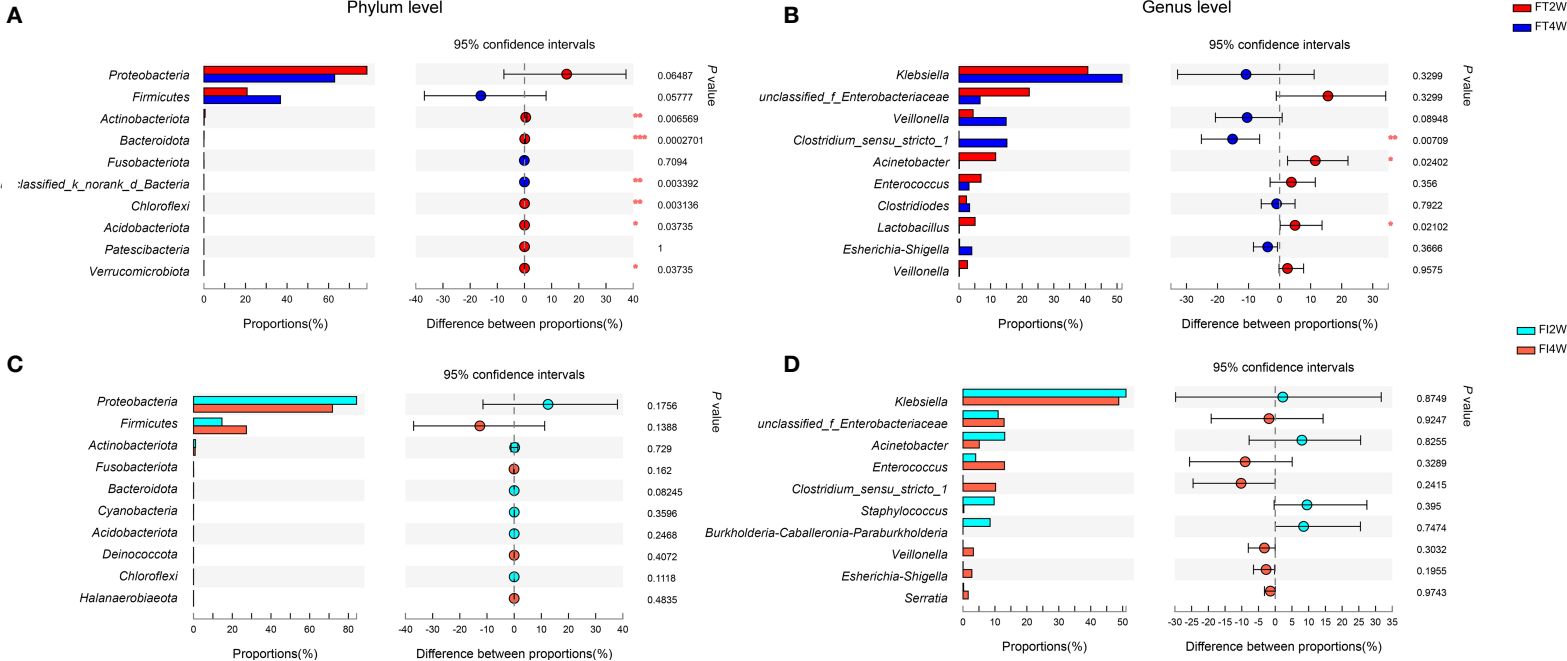
Community abundance within groups. In the FT group, the abundances of *Actinobacteria*, *Bacteroides* and *Chloroflexi* at the phylum level **(A)** and *Clostridium* at the genus level **(B)** were higher at 4 weeks than at 2 weeks of life. The community abundance at the phylum level **(C)** or at the genus level **(D)** was not different between 2 and 4 weeks after birth in the FI group.

Our subsequent comparison of the main microbes between 2 and 4 weeks after birth showed that *Actinobacteria*, *Bacteroides* and *Chloroflexi* at the phylum level and *Clostridium* at the genus level were significantly increased at 4 weeks in the FT group (*P*<0.05), while there were no differences in the FI group (**Figure 4**). Seven communities from phylum to genus at 4 weeks were significantly different from those at 2 weeks in the FT group in the *LEfSe* analysis (LDA score above 4), while none was significantly different in the FI group (**Supplementary Figure S2**).

### SCFAs Measurements

**Figure 5:**
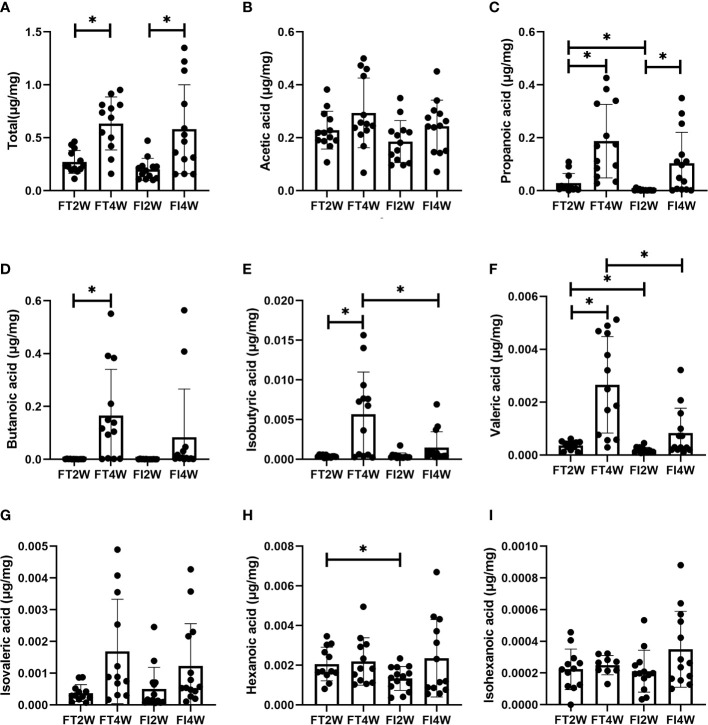
Comparison of concentrations of SCFAs between the FT and FI groups and between 2 and 4 weeks of life. **(A)** Total SCFAs, **(B)** acetic acid, **(C)** propionic acid, **(D)** butanoic acid, **(E)** isobutyric acid, **(F)** valeric acid, **(G)** isovaleric acid, **(H)** hexanoic acid, **(I)** isohexanoic acid. *P < 0.05.

There was no significant difference in the concentration of total SCFAs between the FI and FT groups at 2 and 4 weeks, respectively (*P*>0.05). The levels of propanoic, valeric and hexanoic acids were much lower at 2 weeks in the FI group, and the levels of isobutyric and valeric acids were lower in the FI group at 4 weeks (*P*<0.05) (**Figure 5**).

In the FI group, the concentration of total SCFAs at 4 weeks was higher than that at 2 weeks (*P*<0.05), and we obtained a similar result in the FT group (*P*<0.05). In the FT group, the levels of propanoic, butanoic, isobutyric and valeric acids at 4 weeks were higher than those at 2 weeks, while in the FI group, we found that only the level of propanoic acid was higher (*P*<0.05) (**Figure 5**).

**Figure 6:**
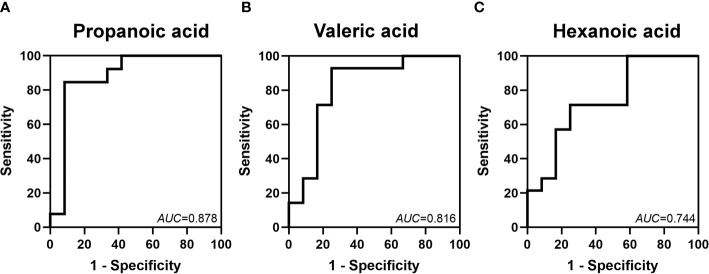
Identification of the value of some SCFAs in the prediction of FI by ROC analysis. The *AUCs* of propanoic **(A)**, valeric **(B)** and hexanoic **(C)** acids at 2 weeks after birth were 0.878, 0.816 and 0.744, respectively.

ROC curves for these metabolites at 2 weeks were constructed to evaluate the value of SCFAs in the early prediction of FI. We found that the *AUCs* of propanoic, valeric and hexanoic acids were 0.878, 0.816 and 0.744, respectively (**Figure 6**).

To further explore the relationship between the gut microbiota composition and SCFAs, heatmap analysis was performed. At the phylum level, dominant *Proteobacteria* abundance was negatively related to the concentrations of butanoic acids and isobutyric acids, and *Firmicutes* abundance was positively related to butanoic acids. At the genus level, *Clostridium* abundance was positively related to butanoic and valeric acids, and *Veillonella* abundance was positively related to propanoic, butanoic, isobutyric and valeric acids. These findings suggested that the concentration of SCFAs varied with the abundance and composition of the gut microbiota (**Supplementary Figure S3**).

### Relative Bioluminescence of AI-2

**Figure 7:**
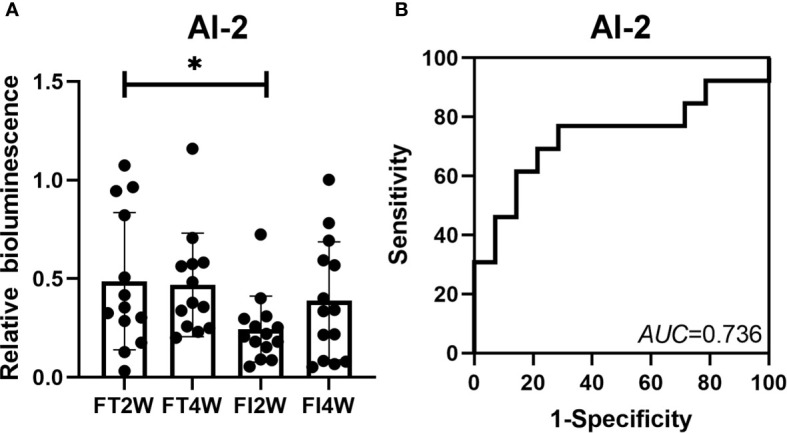
Change in AI-2 level in the FT and FI groups and the value of AI-2 in the prediction of FI. The level of AI-2 was lower in the FI group than in the FT group at 2 weeks of life, and there was no difference between the two groups at 4 weeks after birth **(A)**. The *AUC* for AI-2 at 2 weeks of life was 0.736 **(B)**. *P < 0.05.

The relative bioluminescence of AI-2 was lower in the FI group than in the FT group at 2 weeks after birth (*P*<0.05). However, there was no difference between the two groups at 4 weeks (*P*<0.05). In the FI group, there was no difference in the AI-2 level between 2 and 4 weeks after birth (*P*>0.05), and a similar finding was observed in the FT group. To evaluate the value of AI-2 in predicting FI, a ROC curve analysis of AI-2 at 2 weeks was performed, and the *AUC* was 0.736 (**Figure 7**).

## Discussion

FI is one of the most concerning issues affecting enteral nutrition in preterm infants, and previous studies have shown that dysbacteriosis is a feature of FI (Yuan et al., 2019). In our study, we first compared the diversity and main microbial abundances between the FI and FT groups at 2 weeks of life. We found no difference in the alpha or beta diversity or in the microbiota composition between the FI and FT groups at the phylum and genus levels at 2 weeks after birth. The dominant microbes were *Proteobacteria* and *Firmicutes* at the phylum level and *Klebsiella* at the genus level in both the FI and FT groups. Gut microbiota colonization begins *in utero* and is influenced by many factors, including maternal microbial communities, genetic factors, timing of rupture of the membranes, mode of delivery, postpartum environmental exposures, enteral feeding and the maturity of the infant’s immune system (Underwood and Sohn, 2017). Notably, microbial compositions vary greatly among preterm infants (Simioni et al., 2016; Grier et al., 2017). Therefore, multiple factors might lead to complexity in the composition of the intestinal microbiota in the early postnatal period. The small sample size in this study limits further understanding of the intrinsic characteristics of microbiota changes in the early postnatal period.

In our comparison of diversity and the dominant microbes between the FI and FT groups at 4 weeks after birth, we found that there was no difference in alpha diversity but a large difference in beta diversity. Although there was no difference in the composition of the gut microbiota at the phylum level, *Veillonella* was less abundant in the FI group at the genus level, consistent with a previous study (Li et al., 2021). In our subsequent comparison of the diversity and main microbes between 2 and 4 weeks in both the FI and FT groups, we found that in the FI group, the *Chao* index was lower at 4 weeks, the *Shannon* index was unchanged, and beta diversity had been altered. A similar finding was observed in the FT group. We further found that *Actinobacteria*, *Bacteroides* and *Chloroflexi* at the phylum level and *Clostridium* at the genus level were significantly more abundant at 4 weeks in the FT group, while there were no differences in the FI group. A previous study showed that in infants with NEC, whose clinical features are similar to those of infants with FI, the microbial transition from *γ-Proteobacteria* to *Bacilli* and *Clostridia* was reversed or delayed (Elgin et al., 2016). In our study in infants with FI, we found that the gut microbiota composition changed in a manner similar to that in other studies (La Rosa et al., 2014; Yuan et al., 2019; Li et al., 2021). We speculate that this might be due to gastrointestinal motility disorders that limit the introduction and colonization of gut microbes (Indrio et al., 2011; Gregory et al., 2016), and the microbial composition plays an important role in the recovery of intestinal functions (Jadcherla and Kliegman, 2002; Lin, 2004; Moore and Wilson, 2011). This still needs further study.

SCFAs are generated by *Firmicutes*, *Bacteroides* and *Actinobacteria* in the colon (den Besten et al., 2013). We found that there were large differences in SCFAs concentrations, although there were no differences in the microbiota composition in our study. The levels of propanoic, valeric and hexanoic acids were much lower at 2 weeks than at 4 weeks in the FI group. In the process of intestinal microbiota alterations and human disease, metabolites from microbes often play an important mediating role in host–microorganism interactions (Suez and Elinav, 2017), and changes in their content or concentrations are not always consistent with changes in the microbiota. For example, a study focusing on *Bifidobacterium longum* (BL) in patients with anxiety and irritable bowel syndrome found that although different groups had similar fecal microbiota profiles, the BL group had reduced urine levels of methylamines and aromatic amino acid metabolites (Pinto-Sanchez et al., 2017). Similar findings of inconsistent changes between microbiota compositions and metabolite production were also observed in other studies (Nagpal et al., 2019; Xu et al., 2020). Therefore, this divergent variation between microbiota composition and SCFAs concentrations might also occur in infants with FI, and validation of this finding by further large-sample studies is necessary.

Some microbes, especially *Klebsiella*, are considered potential diagnostic biomarkers for FI (Yuan et al., 2019). To our knowledge, there has been no report on the predictive value of SCFAs in the early diagnosis of FI. We explored the value of SCFAs in the prediction of FI and found that propanoic, valeric and hexanoic acids might be potential biomarkers predictive of FI. As reported previously, SCFAs play important roles in the development of the intestinal tract (den Besten et al., 2013). Propanoic acid increased expression of the tight junction proteins zonula occludens-1 and occludin on the intestinal epithelial barrier in Parkinson’s disease patients and decreased inflammation in a colitis model (Bajic et al., 2020; Huang et al., 2021). Valeric acid improved intestinal epithelial integrity in irradiated mice and reduced the incidence of necrotic enteritis in chickens (Onrust et al., 2018; Li et al., 2020). There are relatively few studies on hexanoic acid in the gut, but it was found that hexanoic acid modulated antimicrobial peptide expression and played an important role in decreasing inflammatory injury (Alva-Murillo et al., 2012). Therefore, propanoic, valeric and hexanoic acids might participate in inflammation and injury processes of the intestine and may reflect the gut condition. We found that the *AUCs* of propanoic, valeric and hexanoic acids were 0.878, 0.816 and 0.744, respectively, in predicting FI at 2 weeks. This finding suggests that they have moderate predictive value (Swets, 1988).

AI-2 is a signaling molecule produced by *LuxS*, an enzyme found in many bacterial species, and has been proposed to promote communication in gram-negative and gram-positive bacteria (Pereira et al., 2013). It plays an important role in the virulence and colonization of pathogenic bacteria (Won et al., 2020). In a previous study, we found that AI-2 might be a new, valuable biomarker for the diagnosis of NEC (Fu et al., 2020), which is a devastating gastrointestinal disease, and some neonates display early symptoms, such as FI in preterm neonates. Based on the close relationship between FI and NEC, we also explored the value of AI-2 in predicting FI in this study and found that AI-2 levels were lower in the FI group than in the FT group at 2 weeks after birth. The *AUC* for AI-2 was 0.736, suggesting that AI-2 had a moderate predictive value in this small-sample-size study, and further large-sample studies are necessary. An interesting finding of this study was that although there was a difference in AI-2 levels between the FI and FT groups, there was no difference in the microbiota composition between the two groups. The findings of our preliminary study on NEC were similar to those of Thompson’s study on microbiota affected by antibiotics (Thompson et al., 2015; Fu et al., 2020); the specific mechanisms need further study.

There were some limitations to our study. We collected fecal samples at 2 and 4 weeks according to clinical features. We found that FI occurred within 2 weeks after birth and that symptoms had disappeared at 4 weeks. The timing of fecal collection needs to be more precise. Additionally, we compared the microbiota composition, SCFAs concentrations and AI-2 levels at only 2 and 4 weeks, so more time points are needed to better understand the changing trends in infants with FI and FT. Although some SCFAs concentrations and AI-2 levels might help in the prediction of FI, clinical verification tests with larger sample sizes need to be conducted in further studies.

## Conclusion

In summary, the main compositions of the microbiota in FI infants at the phylum and genus levels were not obviously different from those in FT infants. Propanoic, valeric and hexanoic acids and AI-2 might have moderate value in predicting FI.

## Data Availability Statement

The datasets presented in this study can be found in online repositories. The names of the repository/repositories and accession number(s) can be found below: https://www.ncbi.nlm.nih.gov/, PRJNA738369.

## Ethics Statement

The studies involving human participants were reviewed and approved by Ethics Committee of the Children’s Hospital Affiliated to Chongqing Medical University (No. 2021-67). Written informed consent to participate in this study was provided by the participants’ legal guardian/next of kin.

## Author Contributions

All seven authors made substantial contributions to the study and manuscript and met the criteria for authorship defined in the author instructions. X-CL collected the clinical data. X-CL, QS, Y-CJ and L-ZF helped collect the fecal samples. X-CL, QS, and Y-CJ worked on basic sample processing and the drafting of the manuscript. Z-LW, YH, and L-QL supervised the project, contributed to the conception and design of the study and analysis and interpretation of the data, and contributed to the critical revision and final approval of the manuscript. X-CL and L-QL performed the final approval of the manuscript.

## Funding

This work was supported by the National Natural Science Foundation of China (82001602), Natural Science Foundation of Chongqing Municipality (cstc2021jcyj-msxmX0063), Joint Medical Research Project of Chongqing Science and Technology Commission (2022MSXM039), Scientific Research Foundation of the Science and Technology Commission of Chongqing (Grant Nos. cstc2019jcyj-msxmX0169) and Science and Health Project of Chongqing Health Commission (2020FYYX217).

## Conflict of Interest

The authors declare that the research was conducted in the absence of any commercial or financial relationships that could be construed as a potential conflict of interest.

## Publisher’s Note

All claims expressed in this article are solely those of the authors and do not necessarily represent those of their affiliated organizations, or those of the publisher, the editors and the reviewers. Any product that may be evaluated in this article, or claim that may be made by its manufacturer, is not guaranteed or endorsed by the publisher.

## Supplementary Material

Supplementary Figure S1
*LEfSe* of the microbiota in FI and FT groups. No communities from phylum to genus were significantly different between the two groups at 2 weeks after birth (LDA score less than 4) **(A)**. Four communities from phylum to genus were significantly different between the two groups at 4 weeks of life (LDA score more than 4) **(B)**.

Supplementary Figure S2
*LEfSe* of the microbiota at 2 and 4 weeks of life. In the FT group, seven communities from phylum to genus at 4 weeks were significantly different from those at 2 weeks (LDA score more than 4) **(A)**. In the FI groups, no communities from phylum to genus at 4 weeks were significantly different from those at 2 weeks (LDA score less than 4) **(B)**.

Supplementary Figure S3 Relationship between the gut microbiota and SCFAs. The change in color reflects the data in the two-dimensional matrix. The color depth indicates the size of the value, and it can intuitively express the size of the value in a defined color depth.
