# Supplementary figures and images for: Differences in the Gut Microbiota Composition and Metabolites Associated With Feeding Intolerance in VLBW Infants With a Gestational Age of ≤ 30 Weeks: A Pilot Study
Source: Front Cell Infect Microbiol. 2022 Feb 17;12:726322. doi: 10.3389/fcimb.2022.726322 (PMC8891543; doi:10.3389/fcimb.2022.726322)

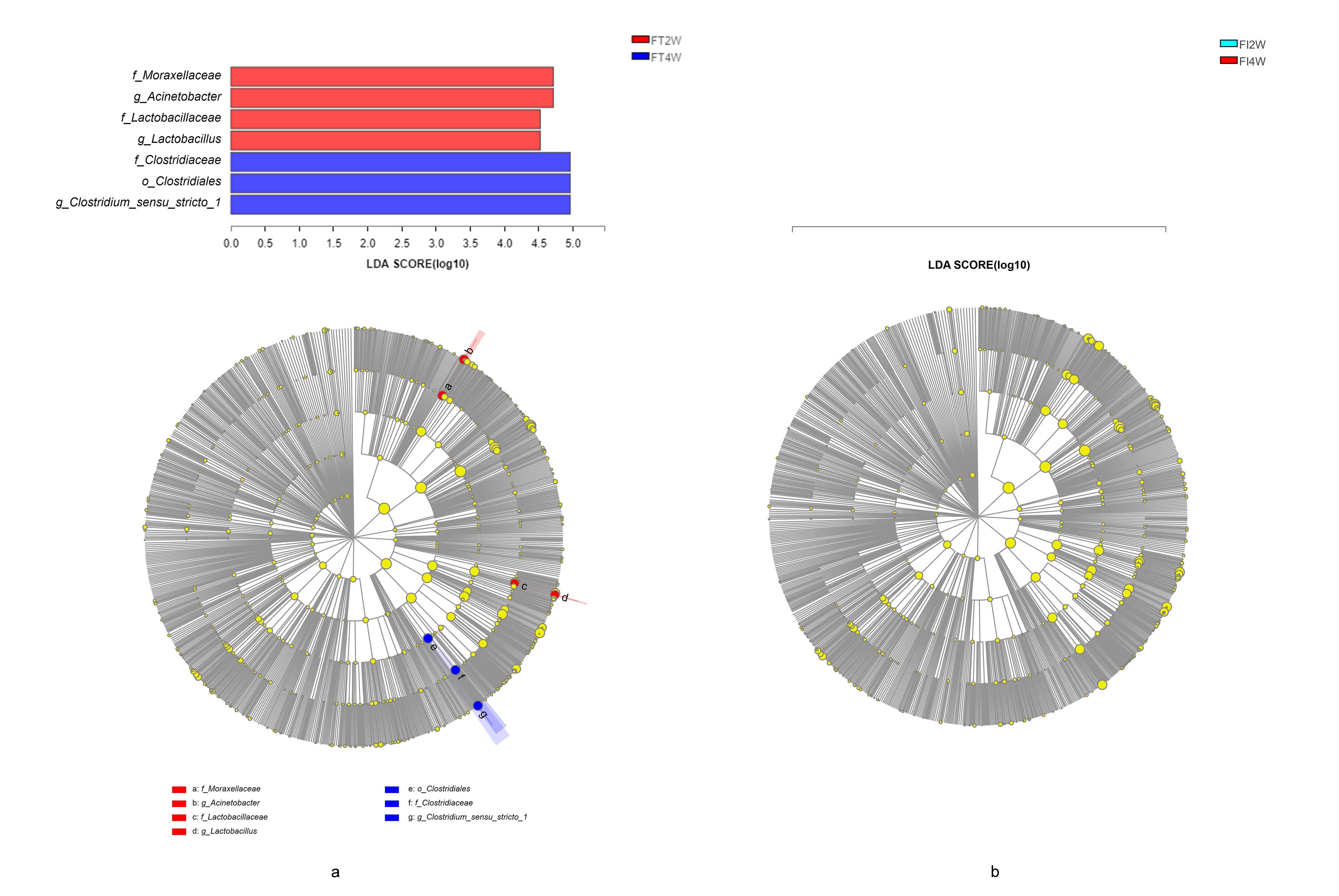

Supplement: Supplementary Figure S2 — LEfSe of the microbiota at 2 and 4 weeks of life. In the FT group, seven communities from phylum to genus at 4 weeks were significantly different from those at 2 weeks (LDA score more than 4) (A). In the FI groups, no communities from phylum to genus at 4 weeks were significantly different from those at 2 weeks (LDA score less than 4) (B). [file Image_2.jpeg]

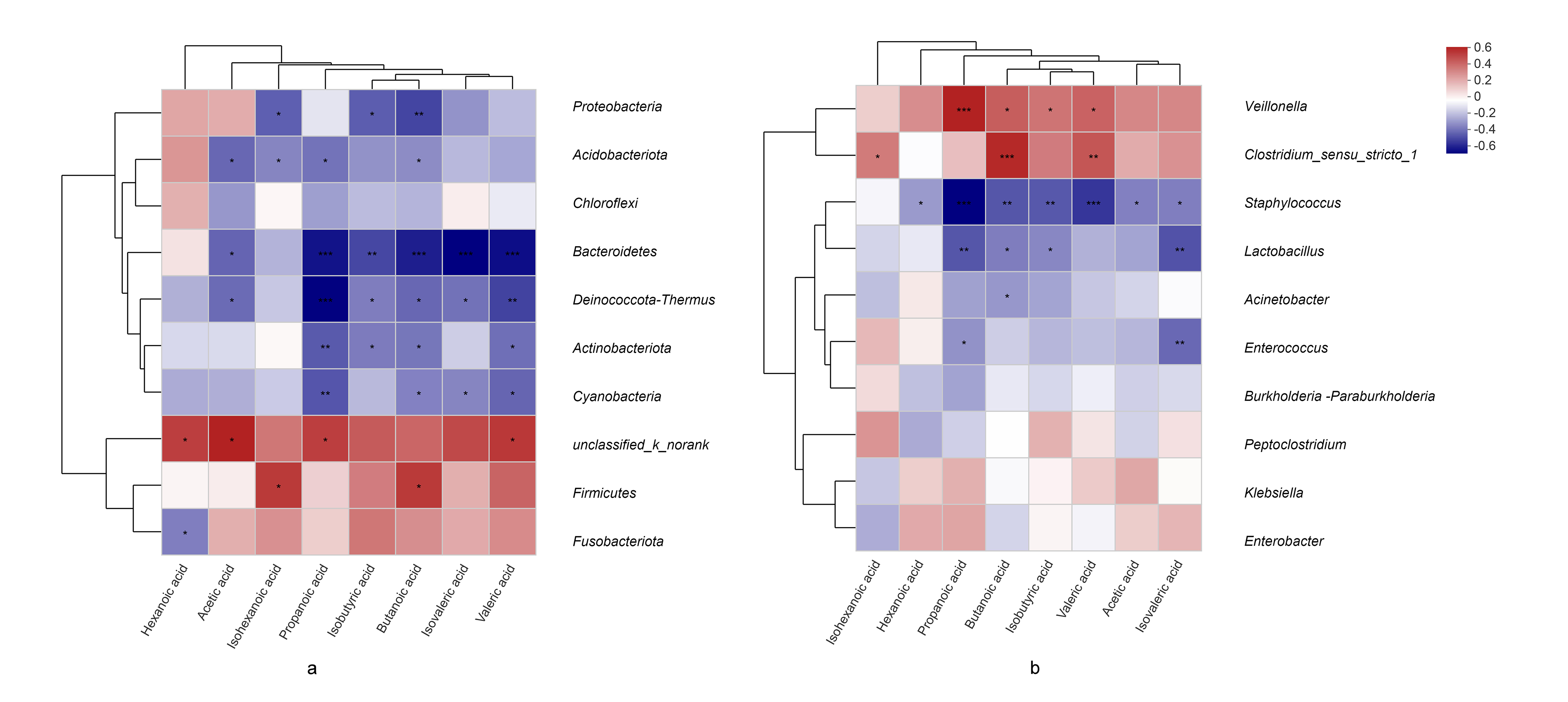

Supplement: Supplementary Figure S3 — Relationship between the gut microbiota and SCFAs. The change in color reflects the data in the two-dimensional matrix. The color depth indicates the size of the value, and it can intuitively express the size of the value in a defined color depth. [file Image_3.jpeg]
